# An Observation on the Particular Conformation of a Foetus, Which Led to Some Difficulty to Determine Its Position in Utero

**Published:** 1803-05-01

**Authors:** 

**Affiliations:** Surgeon at Rouen


### *An* OBSERVATION *on the* PARTICULAR CONFORMATION *of a* FOETUS, *which led to some Difficulty to determine its Position in* UTERO ; *by Cit.* MULOT, *Surgeon at Rouen.*

WHEN the author first saw the patient, he found her attended by a midwife and a surgeon; the child presented the left arm. The author brought down the inferior extremities with much difficulty; and he perceived *the two great toes* were on the outer side, which induced him to think that he held the feet of two distinct children; but on examining which of the two he should first extract, he found that the two members belonged to the same body; he then conceived it probable that the fœtus might be double, or that it might have more than two inferior extremities. When he had passed his fingers somewhat above the pelvis of the child he perceived a large sack filled with water, and which obstructed the passage of the fœtus; he opened this pouch, and the child was born dead without any further difficulty. Some short time before extraction it had given evident marks of life. It appeared, said the author, that this child was not enveloped in its membranes, that the inferior extremities were so changed from their natural position, that the knees were turned towards the sacrum; the pouch, which was opened, covered immediately the intestines; the placenta was very small and the umbilical cord very short. The exterior parts of generation were imperfectly marked, but internally a matrix was to be seen very distinctly.

*Feb.* 22, 1803.

